# Comparative analysis of prevention and control measures toward COVID-19 epidemic between Shanghai and Beijing

**DOI:** 10.3389/fpubh.2023.1121846

**Published:** 2023-04-17

**Authors:** Yueli Meng, Xi Wang, Pei Dong, Yujie Yang, Kun Wang, Xiaoling Yan, Guangyu Hu, Ayan Mao, Wuqi Qiu

**Affiliations:** ^1^Department of Public Health Strategic Information Research, Institute of Medical Information, Chinese Academy of Medical Sciences, Beijing, China; ^2^Department of Science and Education, Institute of Medical Information, Chinese Academy of Medical Sciences, Beijing, China

**Keywords:** COVID-19 epidemic, omicron, epidemic prevention and control, comparative analysis, prevention and control measures

## Abstract

**Purpose:**

By serving and providing a guide for other regional places, this study aims to advance and guide the epidemic prevention and control methods, and practices and strengthen people’s ability to respond to COVID-19 and other future potential public health risks.

**Design/methodology/approach:**

A comparative analysis was conducted that the COVID-19 epidemic development trend and prevention and control effects both in Beijing and Shanghai. In fact, regarding the COVID-19 policy and strategic areas, the differences between governmental, social, and professional management were discussed and explored. To prevent and be ready for potential pandemics, experience and knowledge were used and summarized.

**Findings:**

The strong attack of the Omicron variant in early 2022 has posed challenges to epidemic prevention and control practices in many Chinese cities. Shanghai, which had achieved relatively good performance in the fight against the epidemic, has exposed limitations in its epidemic prevention and control system in the face of Omicron. In fact, the city of Beijing has undertaken prompt and severe lockdown measures and achieved rather good results in epidemic prevention and control because of learning from Shanghai’s experience and lessons; adhering to the overall concept of “dynamic clearing,” implementing precise prevention and monitoring, enhancing community control, and making emergency plans and preparations. All these actions and measures are still essential in the shift from pandemic response to pandemic control.

**Research limitations/implications:**

Different places have introduced different urgent policies to control the spread of the pandemic. Strategies to control COVID-19 have often been based on preliminary and limited data and have tended to be slow to evolve as new evidence emerges. Hence, the effects of these anti-epidemic policies need to be further tested.

## 1. Introduction

It has been two and a half years since the outbreak of COVID-19 at the end of 2019, and it is still prevalent world widely. In China, prevention and control of the epidemic has always been under pressure (1, 2). Shanghai is the most populous urban area in China with 40 million inhabitants living in the Shanghai metropolitan area and the only city in East Asia with a GDP greater than its corresponding capital. With the characteristics of a large population, a high degree of agglomeration, growing social mobility, and frequent export and import, the prevention and control of the epidemic are under great pressure from both inside and outside (3). As of December 31, 2021, Shanghai has effectively and accurately balanced the relationship between the fight against the COVID-19 epidemic, economic and social development, and normal life and production. Pursuing the concept of “balanced anti-epidemic” and adopting the prevention and control model of “professional governance, precise prevention and control, and lean execution.” The risk and cost of the epidemic have been relatively reduced (4). However, with the latest severe acute respiratory syndrome coronavirus 2 (SARS-CoV-2) variant Omicron (B.1.1.529) has been revealed that it is over ten times more contagious than the original virus or about twice as infectious as the Delta variant. The “balanced anti-epidemic” policy shows its limitations. At the beginning of March 2022, Shanghai experienced the largest cluster of infections since 2020, which lasted for nearly four months. Subsequently, at the end of April, a cluster of outbreaks caused by Omicron also broke out in Beijing, but the number of infections was significantly reduced. These epidemic clusters have highlighted several issues and gaps in China’s present epidemic prevention and control measures. In many cases, fragmentation between public health on the one hand, and political and economic priorities on the other, has led to confusion in reaching policy decisions about how to control the pandemic, preserve lives, avoid social disruption, and protect the economy. Not only this paper aims to further improve the health policies and guidelines for other regions to prevent and prepare for future pandemics, but it also summarizes the development trend of epidemics in Shanghai and Beijing from March to June 2022. Plus, the literature regarding prevention and control measures from Shanghai and Beijing are consulted and summarized as well. The data in this article was gathered from open reports, pertinent literature, and the local health committee’s official website. Open reports typically refers to government reports, academic papers, and news articles which published online and can be accessed by anyone. Pertinent literature refers to relevant academic or scientific studies, publications, or research in the field. This include studies that have been conducted by experts in the subject matter, peer-reviewed articles, and books. Local health committee’s official website refers to local government agencies or non-governmental organizations responsible for health-related matters. By gathering information from these multiple sources, a comprehensive and accurate account of the topic at hand can be provided to support claims.

## 2. Overview of the epidemics in Shanghai and Beijing

### 2.1. Development trend of the epidemic in Shanghai

**Figure 1:**
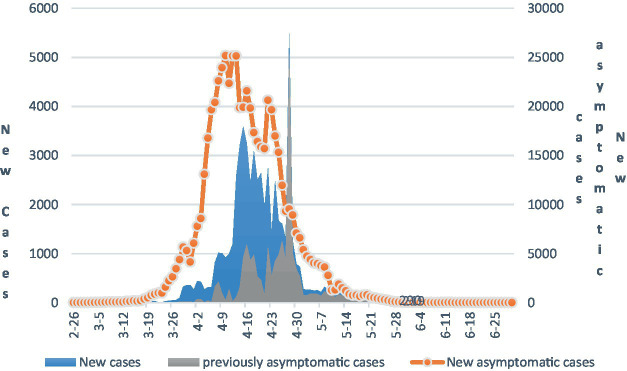
The development of the COVID-19 epidemic in Shanghai.

From February 26, 2022, to June 30, 2022, a total of 649,662 local cases of infections were reported in Shanghai, including 58,137 confirmed cases, 591,525 asymptomatic infections, and 588 deaths.^1^ Judging from the development trend of the current round of the COVID-19 epidemic in Shanghai, which is shown in Figure 1 and the data are available that the changes in the number of new confirmed cases and asymptomatic infections of the epidemic in Shanghai first remained at a low level, then increased rapidly, and then gradually decreased after a period of fluctuation. Since June 1st, Shanghai has maintained a single-digit or low level of new confirmed cases and new asymptomatic infections for one month in a row. The current round of the epidemic in Shanghai lasted for nearly 4 months.

Details on the prior Shanghai Municipal Health Commission News Center Daily News Release Statistics report can be found at: https://wsjkw.sh.gov.cn/xwfb/20220701/701ab5f0a08d4327ab4174d1ce.

### 2.2. Development trend of the epidemic in Beijing

**Figure 2:**
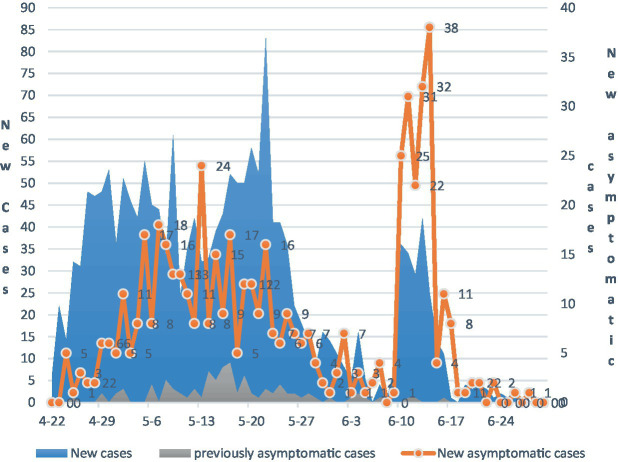
The development of the COVID-19 epidemic in Beijing.

From 00:00 on April 22, 2022, to 24:00 on June 30, 2022, Beijing had reported a total of 2,325 community cases of his COVID-19 epidemic, including 1788 confirmed cases of his and 537 asymptomatic cases, infected persons are indeed included. Among those infected, 69.38% had mild disease, 7.48% had a common disease, and 23.10% had an asymptomatic infection. There was only one severe patient and no critical illness or death.^2^ Judging from the development trend of the current round of COVID-19 in Beijing, which can be shown in Figure 2, the COVID-19 pandemic is generally characterized by a fluctuating increase; throughout the analysis period, the number of new confirmed cases and new asymptomatic infections was in low increase, and the proportion of previously asymptomatic infections turned into confirmed cases was low (5.26%). Several peaks are related to several large-scale clustered epidemics in the corresponding time period.

### 2.3. Analysis of the spread characteristics of the epidemic

The COVID-19 epidemic in the current round in Shanghai and Beijing was sequenced by the two cities’ CDC virus gene sequencing results, and it was shown that they were all Omicron variant strains (most of them in Shanghai were Omicron BA.2 and BA). 2.2, which has the characteristics of a short incubation period, rapid transmission, strong infectivity, strong concealment, and immune escape (5). Studies have shown that the R0 (basic reproduction number) of Omicron BA.1 is 9.5, the transmission ability of BA.2 is 1.4 times that of BA.1, and R0 is about 13.3 (6).

This round of epidemic in Shanghai is the most severe one since then. In the early stage of the epidemic, the source of infection was focused on the contamination of the environment by the virus carried by imported cases from other countries, which caused local infection (7), resulting in multi-chain parallel, multi-point community transmission, the high incidence in local areas, and rapid spread. The following are the key symptoms: First, a substantial percentage of reported infections (91.1%) are asymptomatic infections, which are more latent and difficult to detect. Second, the current outbreak is extensive, affecting a variety of metropolitan populations from different racial and ethnic backgrounds, and the chain of transmission is intricate. Third, there are more people entering the country and more cases being imported, there are more investigative assistance missions being sent to national regions, and local epidemics are entwined and overlapped. a significant impact on the city’s isolation. Housing options and medical care (8). The current round of the epidemic in Beijing presents the characteristics of rapid spread, multiple points and wide areas, and there are sporadic hidden sources of infection in society. The Beijing Center for Disease Control and Prevention analyzed the cases that caused more secondary infections since April 22 and found the following characteristics: First, there were lots of gatherings during the infectious period, which led to clustered epidemics; second, those who are unvaccinated are more likely to contract an illness; third, the range of activities is greater, which causes the epidemic to spread to various locations. Compared with the epidemic in Shanghai, the early infections in Beijing’s current round of epidemics originated from the spillover of clustered epidemics outside Beijing, and the transmission chain is relatively clear (9); the number of asymptomatic infections is small (23.1%), and most of the infected cases (93.1%) were discovered during isolation control, and social screening cases accounted for only 6.9%.

## 3. Summary of public health epidemic control measures in Shanghai and Beijing

According to a “conceptual model of major epidemic crisis governance system and mechanism” based on theories of crisis management and collaborative governance, which was constructed by some scholars, this article summarizes the public health epidemic prevention and control strategies and measures from this round of epidemic in Shanghai and in Beijing. The difference in government management, social management, and professional management concerning COVID-19 policy and strategic areas was discussed in detail.

### 3.1. Epidemic control measures in Shanghai

#### 3.1.1. Government governance perspective

Nowadays, the COVID-19 pandemic is still the biggest uncertainty affecting the world economy. On March 17, according to General Secretary Xi Jinping’s speech at the meeting of the Standing Committee of the Political Bureau of the CPC Central Committee that striving to achieve the greatest effect of prevention and control at the lowest cost and minimize the impact of the epidemic on economic and social development, it shows the determination and confidence in coordinating epidemic control strategies and economic and social development (10). Non-pharmaceutical interventions such as lockdown management are effective in reducing the infection among close contacts and frontline healthcare workers to minimize the spread of the virus in the community and even contain the pandemic (11). How to balance epidemic prevention measures and economic development poses a huge challenge for decision-makers. As one of the megacities in China, Shanghai plays a significant role in national economic development and technological innovation (3). Some scholars use high-frequency truck flow data between cities to estimate the impact of the closure on the city’s real income and the resulting spillover effects. The results of the study show the impact on the total real income of implementing a full-scale lockdown for one month in each city, with the three most affected cities being Shanghai, Beijing, and Shenzhen, where a full-scale lockdown would reduce total real income by 2.7% in each of these cities, 2.5 and 1.8% (11). Meanwhile, after the closure of Shanghai, factories in the city shut down, commerce was static, consumption decreased, and tax revenue suffered huge losses. In addition, core technologies of many industries such as chips, semiconductors, new energy vehicles, and artificial intelligence are in Shanghai. After the city closed, all upstream and downstream enterprises in the supply chain were affected. Considering the contradiction between epidemic prevention measures and economic development, coupled with the insufficient understanding of Omicron in the early stage (12), At the start of this outbreak, Shanghai did not take any precautions to shut down the city, which allowed the Omicron mutant strain to spread covertly. Rapid growth in the number of asymptomatic infections has created precise prevention and control challenges.

#### 3.1.2. Social governance perspective

The large-scale infection of the Omicron strain has exposed many problems from the perspective of social governance. One is the issue of emergency supplies. The outbreak of the epidemic has had a sudden impact on the supply chain of many commodities, especially the emergency supplies that are closely related to the epidemic, which is often in short supply under the circumstances of growing demand and limited supply (13). Emergency material security is an important basis for coordinating economic development, ensuring people’s livelihood, and preventing and defusing major risks (14). The attack of this round of Omicron mutant strains has exposed Shanghai’s relatively lagging in terms of emergency material reserves such as limited storage capacity, single stock varieties, unreasonable division of blocks, and emergency logistics and poor transportation (14), resulting in chaotic situations such as difficulties in ensuring the supply of living materials for residents and driving up prices in the early stages of this round of epidemic. The second is the shortage of medical resources and the breakdown of manpower. Due to medical fragility and the destruction of the balance of medical resources under sudden outbreaks (15), designated hospitals, shelter hospitals, or centralized isolation sites were operating at full capacity during the high-level operation of this round of epidemic in Shanghai, and medical staff and medicines were insufficient; Large-scale test for COVID-19 has raised the demand on the number and capacity of personnel from the community, volunteers, medical institutions, and testing institutions (16). At the same time, the lack of professional facilities and capacity building in the infection wards of some general hospitals has brought challenges to the routine medical treatment and emergency service needs of critical patients with chronic diseases (17). The third is related to primary care. Primary care should be the backbone of any healthcare system. It is community-based (18). At present, the resident population of Shanghai has exceeded 20 million, but the scale of the community is still limited and the relationship between power and responsibility is not equal. As a result, the capacity of the community is insufficient which is unable to respond to the needs of large-scale epidemic control. It should also take a lot of time to link up the actions of various parties, such as medical groups, supply guarantee units, property management, and the government (19).

#### 3.1.3. Professional governance perspective

Professional governance is the technical dimension of Shanghai’s “balanced anti-epidemic” model. Before this round of epidemic, Shanghai’s relatively successful anti-epidemic was based on professional measures against virus transmission (4). However, in the face of this round of sudden outbreaks, Shanghai did not fully follow medical knowledge and scientific strategies and did not take decisive measures in the early stages of the outbreak. As a result, the current round of epidemics had spread in Shanghai for more than a month before large-scale lockdown measures were implemented, resulting in widespread community dissemination. It can be seen that improving the ability of epidemic prevention and early detection, timely diagnosis and adopting movement restriction measures are effective public health interventions for the prevention and control of Omicron (5). In addition, the current outbreak of the Omicron epidemic has exposed the lack of effective emergency plans in society. Professionals had predicted that the epidemic would come in waves and would even have larger-scale outbreaks than the previous ones before the current round of outbreaks in Shanghai. However, in the early stages of the outbreak, Shanghai lacked a set of general social contingency plans to adhere to, and most of the temporary plans were poorly executed, squandering resources, and delaying possibilities for the response. For instance, the government has prepared over 1,000 supply guarantee firms for material dispatch after people remain at home, but these temporary supply guarantee companies are unequal. Large enterprises that already have a complete set of logistics systems have not tried their best to mobilize them, and there is no contingency plan for the mobilization of social resources (13). At the same time, there are governance shortcomings such as insufficient anti-epidemic norms, unbalanced development, limited emergency response capabilities, and unsound coordination and cooperation mechanisms in community participation in epidemic prevention (20).

### 3.2. Epidemic control measures in Beijing

#### 3.2.1. Government governance perspective

Based on the knowledge and preventative strategies learned from Shanghai’s epidemic prevention and control, policymakers swiftly reach a consensus. The epidemic prevention and control authorities in Beijing have a thorough understanding of the prevention and control policies established by the CPC Central Committee based on the experience and control measures from Shanghai, unwaveringly adhere to the general policy of “dynamic clearing,” and strictly and fervently follow it considering the strong infectiousness, rapid transmission, high proportion of asymptomatic infections, and strong occult transmission of the Omicron. From the beginning of the outbreak, Beijing’s leaders adopted a hard political stance. Plus, different departments understood the relevant policies and clarified their respective tasks (21).

#### 3.2.2. Social governance perspective

First, all citizens in Beijing participated in the whole process of epidemic prevention and control. The “four-party responsibility” system of territories, departments, units, and individuals should be fully implemented, the whole people should be mobilized and the whole city should be controlled, and it should be clarified that any unit, enterprise, and individual is responsible for assisting, cooperating, and obeying the prevention and control work organized by government departments under self-protection, otherwise they will be held accountable in accordance with the law (21).

For instance, the business license for the Paradise Supermarket bar outbreak on June 9 has been canceled, and the appropriate parties responsible have been permitted to be arrested. The second is to implement several safeguards to guarantee various daily demands. Several steps have been actively implemented to assure the market supply of daily requirements in the capital during the implementation of the epidemic prevention and control activities, including stable trading of refined grain and oil products and adequate reserves. According to the Beijing Municipal Bureau of Commerce, the supply of various daily necessities in Beijing is sufficient and stable, and the supply is guaranteed. Beijing attaches great importance to the construction of an emergency material support system. In addition to the government’s storage of strategic materials, it also entrusts commercial enterprises to store a large number of daily necessities (22). The third is to strengthen community governance, forming a grid-based management team composed of government officials, volunteers, and other community workers. The mechanism of community consultative democracy has been continuously deepened, and a multi-dimensional co-governance community should be carried out, forming a grassroots governance structure involving the participation of neighborhood committees, owner committees, property management, volunteers, and residential units, etc. (18). A successful partnership between CDC and the community was established to assist people with functional needs. Community systems for medication and information distribution were fully used.

#### 3.2.3. Professional governance perspective

Compared with Shanghai, the prevention and control authorities in Beijing made decisive decisions in the early stage of this round of the COVID-19 epidemic. The speed and efforts of the prevention and control in Beijing far exceeded those in the previous outbreaks of the original strain of COVID-19, Alpha, and Delta. Since the first new case in the COVID-19 outbreak on April 21, the epidemic prevention authorities found cases in Huairou, Pinggu, and other scenic spots through investigation. The epidemic prevention authorities quickly carried out testing around the scenic spots. On April 22, 15 new confirmed cases were reported in Pinggu District, Dongcheng District, Chaoyang District, Shunyi District, and other areas. The scope and risk of virus transmission increased. By April 23, it was found that there were confirmed cases involving the schools, and the prevention and control authorities were aware of the possibility of multiple outbreaks. At 5 PM on the 23rd, the affected portions of Panjiayuan Street have swiftly declared a closed area and a restricted area for strict monitoring to stop the epidemic’s unchecked spread. The prevention and control authorities have put plans in place to halt the epidemic’s spread as quickly as possible. There was widespread screening, testing, and prompt contact tracing. Early, forceful action promptly and successfully controlled the infection source and broke the chain of epidemic transmission.

## 4. Discussion

### 4.1. Heighten ideological understanding and adhere to the general policy of “dynamic clearing”

The continuous mutation of the COVID-19 strain has made precise prevention and control more difficult. Some studies have shown that although the fatality rate during the epidemic of the Omicron variant did decrease, the total number of deaths caused by the epidemic during the same period was higher than that of the Delta variant, and the harm of Omicron epidemic was still serious (23). China has a large population with a large elderly population, unbalanced regional development, and insufficient medical resources. The relaxation of prevention and control measures will inevitably lead to large-scale infection, severe illness, and death. Economic and social development and people’s health will be seriously affected. We must have a deep, complete, and comprehensive understanding of the guidelines and policies for epidemic prevention and control.

### 4.2. Optimize prevention and control strategies, and strengthen precise prevention and control, and monitoring

Because of the characteristics of the Omicron mutant strain, on the one hand, it is necessary to strengthen the rapid, decisive, and thorough adoption of relevant prevention and control measures, and at the same time continuously optimize and improve prevention and control strategies, strengthen precise scientific prevention and control, and strictly implement normalized epidemic prevention and control measures, implement the requirements of human, physical and environmental coordination (24). The review conducted by Ren demonstrate that Omicron is highly transmissible and faster spread, hence early prevention like vaccination should be taken into consideration, which is consistent with our findings (25). We should balance the relationship between epidemic prevention and control, social and economic development, and normal production and life to the greatest extent. Monitoring is an extremely effective tool for early detection. If the monitoring is done correctly, the sooner the patient is located, the sooner we can act. To minimize the spread of the outbreak, we’ll make sure it’s identified and addressed as soon as possible. We will enhance all aspects of the early warning and multi-channel monitoring systems, as well as the monitoring, early warning, and emergency response capabilities related to epidemics on the other side, it is essential to enhance the COVID-19 normalized testing mechanism, support regional screening, further improve the design of sampling locations, and satisfy public demand for formalized testing. It is necessary to do a good job in the monitoring and management of key populations, key places, and key institutions. Molecular tests such as PCR, as one of the major methods for the detection of SARS-CoV-2 infection, have proven to be crucial to the COVID-19 pandemic response. Antigen rapid detection tests detect viral proteins and, although they are less sensitive than molecular tests, have the advantages of being easier to do, giving a faster time to result, being lower cost, and being able to detect infection in those who are most likely to be at risk of transmitting the virus to others (26). It is necessary to make good use of the effective combination of antigen screening and testing and diagnosis as a monitoring method to detect infected people as early as possible and prevent them from happening. It is also necessary to prevent missed detection caused by irregular sampling due to problems such as insufficient manpower, overloaded work, inadequate protective measures, and insufficient protective awareness, failing to detect potential cases in time (27). All these measures continue to have a crucial role in the transition from pandemic response to pandemic control.

### 4.3. Strengthen community prevention and control, and control the spread of the epidemic as soon as possible

There are two basic fronts in COVID-19 epidemic prevention and control, one is patient treatment, and the other is community prevention and control (28). Communities are densely populated, complex, and have a large flow of people. They play a significant role in joint prevention and control of the epidemic, and can effectively defense by preventing the import from outside and the spread from inside (27). Effectively implement the responsibilities of the four parties, give full play to the roles of neighborhood committees, villagers’ committees, public health committees, and volunteer organizations, organize government officials to effectively sink into the community, implement divisional contracting, and make good use of township health centers, community service centers, and village clinics in the community. In the handling of the epidemic, it is necessary to be more efficient and coordinated, to ensure the mutual coordination of testing, epidemiological investigation, quarantine and treatment, and community control. Various systems and various types of information should be interconnected to ensure that the community is informed in time. Peronace also claimed in her research that prompt information sharing among global public health partners should be significant in pandemic prevention and control (28). We should take more practical and thoughtful measures to ensure the work and livelihood of front-line staff, and allocate human resources appropriately. A successful partnership between CDC and the community should be established to assist people with functional needs. Community systems for medication and information distribution can be fully used (29).

### 4.4. Make emergency plans and preparations, strengthen training mechanism

To achieve good results in epidemic prevention and control, in addition to the patient treatment and community prevention and control, the full participation of the whole people and society is still needed. In the wake of the lockdown, many cities have experienced inadequate public services, such as weak epidemic prevention and control, chaotic social order, and lack of material support. Facing the emergency, the entire society lacks effective emergency plans. After SARS, China established a “one case, three systems” emergency management system with Chinese characteristics. The emergency plan of the pertinent medical and health departments has also been revised and improved considering the COVID-19 epidemic test, but other pertinent departments of epidemic prevention and control, such as production, living, education, recreation, transportation, and public services, should also make the necessary emergency preparations. Different epidemic scenarios should be addressed by emergency plans, and essential employees should also undergo more training and drills. Consolidate primary responsibilities, strengthen the normalized prevention, and control of key locations, and implement comprehensive prevention and control strategies and emergency plans. Adhere to the problem-oriented approach to strengthen prevention and control preparations, strengthen regional defense assistance, and provincial overall planning, and take multiple measures simultaneously. Strengthen shelter hospitals, designated hospitals, centralized isolation points, and related prevention and control materials. During the epidemic prevention and control period, we must also make preparations and related plans for how to ensure the normal life of people in the community (30–34). Since the coronavirus pandemic represents a worldwide health emergency, coordinated actions are required to address it. The epidemic prevention and control strategies used in Beijing and Shanghai serve as a model for other places, helping us to better prepare for COVID-19 and other potential risks to public health in the future. As previously said, it is advised to follow the general strategy of “dynamic clearance,” put precise prevention and monitoring in place, improve community control, and develop emergency plans and preparations. All these measures continue to have a crucial role in the transition from pandemic response to pandemic control.

## 5. Conclusion

From the above discussion, the conclusion can be reached that by serving and providing a guide for other regional places, this study set out to advance and guide the epidemic prevention and control methods, and practices and strengthen people’s ability to respond to COVID-19 and other future potential public health risks. A comparative analysis was conducted that the COVID-19 epidemic development trend and prevention and control effects both in Beijing and Shanghai has extended our knowledge of the differences between governmental, social, and professional management were discussed and explored, regarding the COVID-19 policy and strategic areas. The insights gained from this study may be of assistance to prevent and be ready for potential pandemics. This study has found that generally that Shanghai, which had achieved relatively good performance in the fight against the epidemic, has exposed limitations in its epidemic prevention and control system in the face of Omicron. In fact, the city of Beijing has undertaken prompt and severe lockdown measures and achieved rather good results in epidemic prevention and control because of learning from Shanghai’s experience and lessons; adhering to the overall concept of “dynamic clearing,” implementing precise prevention and monitoring, enhancing community control, and making emergency plans and preparations. All these actions and measures are still essential in the shift from pandemic response to pandemic control. Different places have introduced different urgent policies to control the spread of the pandemic. Strategies to control COVID-19 have often been based on preliminary and limited data and have tended to be slow to evolve as new evidence emerges. Hence, the effects of these anti-epidemic policies need to be further tested. This would be a fruitful area for further work.

## Data availability statement

The original contributions presented in the study are included in the article/supplementary material, further inquiries can be directed to the corresponding author.

## Author contributions

YLM analyzed and interpreted the data, designed the study, and performed the research. XW was a major contributor in writing the manuscript. AYM and WQQ developed the idea for the study and provided supervision. PD, YJY, and GYH conceived the idea and helped collect the data. KW and XLY contributed to the revisions. All authors listed have made a substantial, direct, and intellectual contribution to the work. They have read and approved the final manuscript for publication.

## Funding

The study was funded by Key Project of Decision-making Consultation of Beijing Social Science Foundation, 22JCB041, study on improving the system and mechanism for regular epidemic prevention and control in Beijing.

## Conflict of interest

The authors declare that the research was conducted in the absence of any commercial or financial relationships that could be construed as a potential conflict of interest.

## Publisher’s note

All claims expressed in this article are solely those of the authors and do not necessarily represent those of their affiliated organizations, or those of the publisher, the editors and the reviewers. Any product that may be evaluated in this article, or claim that may be made by its manufacturer, is not guaranteed or endorsed by the publisher.

## Footnotes

^1^Data source: Daily news release statistics of Shanghai Municipal Health Commission News Center. https://wsjkw.sh.gov.cn/xwfb/20220701/701ab5f0a08d4327ab4174d1ce.

^2^Data source: Venezuelan news releases statistics daily. http://wjw.beijing.gov.cn/xwzx_20031/wnxw/index.html.
